# Analysis and Modeling Methodologies for Heat Exchanges of Deep-Sea In Situ Spectroscopy Detection System Based on ROV

**DOI:** 10.3390/s18082729

**Published:** 2018-08-20

**Authors:** Xiaorui Liu, Fujun Qi, Wangquan Ye, Kai Cheng, Jinjia Guo, Ronger Zheng

**Affiliations:** Optics and Optoelectronics Laboratory, College of Information Science and Engineering, Ocean University of China, Qingdao 266100, China; liuxiaorui@stu.ouc.edu.cn (X.L.); jxyewaqu@163.com (W.Y.); chengkai@ouc.edu.cn (K.C.); opticsc@ouc.edu.cn (J.G.); rzheng@ouc.edu.cn (R.Z.)

**Keywords:** remotely operated vehicle (ROV), autoregressive exogenous model, profile measurement, model-based prediction, Raman spectroscopy

## Abstract

In recent years, cabled ocean observation technology has been increasingly used for deep sea in situ research. As sophisticated sensor or measurement system starts to be applied on a remotely operated vehicle (ROV), it presents the requirement to maintain a stable condition of measurement system cabin. In this paper, we introduce one kind of ROV-based Raman spectroscopy measurement system (DOCARS) and discuss the development characteristics of its cabin condition during profile measurement process. An available and straightforward modeling methodology is proposed to realize predictive control for this trend. This methodology is based on the Autoregressive Exogenous (ARX) model and is optimized through a series of sea-going test data. The fitting result demonstrates that during profile measurement processes this model can availably predict the development trends of DORCAS’s cabin condition during the profile measurement process.

## 1. Introduction

Cabled ocean observation technology is gradually becoming an effective tool for ocean research. It provides abundant power and bandwidth communication channels for underwater sensors and relevant systems and supports them to carry out real-time studies on ocean processes across a long time period [1]. Compared with traditional research methods that are limited in endurance and loading, the cabled ocean observation platform has abundant mechanical and electric payload to integrate more sophisticated sensor or measurement systems [2,3]. Take Raman spectroscopy measurement technology for instance, Raman spectroscopy measurement technology can measure the target substance of solid, liquid, and gas phases, thereby greatly extending the range of research. It also has the capability to measure small samples and provides geochemical traverses across a heterogeneous sample [4,5,6]. Since the first deep ocean Raman in situ spectrometer (DORISS) was sent down to the seafloor at a depth of 3600 m in 2004 [4], a relevant measurement system has been widely applied in the cabled observation field [7,8,9,10]. Present progress in deep sea Raman spectroscopy technology has enabled measurement for multi-element molecules (CH_4_, SO_4_^2−^, H_2_S, HS^−^, etc.) in seafloor spices of different phases. Multiple chemical distributions across the seawater profile can also be recorded for further analysis [11,12].

Currently, there are basically two kinds of cabled observation platforms, seafloor observatory network and remotely operated vehicle (ROV). Deep sea Raman measurement system both used to mount and successfully apply on these two platforms. The seafloor observatory network is one kind of relatively stationary system [13]. The example observatory system is the North East Pacific Time-Integrated Undersea Networked Experiments (NEPTUNE) [14,15] and South China Sea observatory network [16,17]. Compared to the observatory network, ROV is more flexible ship-based cabled observation platform. Although its operating period of a single task is less than the observatory network, during this process it can cruise on the seafloor and measure spots (hydrothermal vent, cold seep filed, and so on) and specific vertical seawater layers that the operator is interested in [4]. The mechanical arm and other actuator devices of ROV also make it possible to carry out complex experimental operations on in situ target species of interest [18]. On the other hand, ROV, as a real-time platform, has more flexibility in profile measurement process than subsurface buoys. Based on the above characters, ROV has become an ideal platform to integrate multiple measurement systems and execute measurements for the deep sea environment [19]. In this work, one newly developed ROV-based Raman spectroscopy measurement system (Deep Ocean Compact Automatic Raman Spectrometer, DOCARS) is introduced. It was deployed on the remotely operated vehicle Faxian and on the research vessel Kexue, and successfully executed measurement of the seawater hydrothermal field and seawater profile (at Southwest Pacific). As increasingly sophisticated devices are integrated into the in situ measurement system, it unavoidably presents more requirements for systematic stability. Take Raman spectroscopy for example, high-precision optical devices usually are sensitive to temperature, humidity, and pressure of the system cabin [7]. ROV research tasks usually comprise of two stages, one is the profile measurement stage and the other is the seafloor cruising stage. In this process, the measurement system on ROV needs to ensure not only perennially cold (274–276 K) in seafloor [20,21], but also temperature gradient in seawater profile. For the in situ measurement system, it has to ensure temperature changes from 303 K to 275 K during the deep diving process (with depth of 2000 m) [22], which may cause frequency shift and calculation error [23,24] of spectrum data. So, it will be useful to build a straightforward model to predict the system cabin condition development trend during profile detection [25].

Currently, there is little research on the modeling methodology of condition inside deep sea in situ measurement system. G. McDonald et al. [26] and O. Karim et al. [27] tried to build a model to simulate the heat balance of the junction box of the seafloor observatory network. These models could quantify temperature distribution inside junction box when seawater temperature is stable, but it cannot apply to the profile condition that has a dynamic and complex temperature distribution. Nasr, K. Ben et al. [28] proposed a numerical simulation method for specific mechanical structure, but this method is unable to simulate heat exchange during profile process because material character of DOCARS’s shell contradict with boundary condition of his method.

In this paper, we firstly describe the design of DOCARS, then a novel modeling methodology is discussed to realize and predict the cabin condition development when DOCARS is in the profile measurement process. This methodology is built based on Autoregressive Exogenous (ARX) prediction model and optimized with data abstracted from practical sea-going research. The result of the fitting degree between simulation and measured data indicates the availability of this modeling methodology.

## 2. System Design

DOCARS was equipped with several modules, including the adapter, optical load, and feedback unit. The adapter includes underlying hardware such as an Ethernet switch, power supply circuit, and relay protection module. The feedback unit includes varieties of sensors to provide the ship-based terminal with system status information (such as condition parameters and electric parameters). The optical load includes a customized high-sensitivity 532nm Laser (AUT-FSRL-532-300T, Aunion Tech Co., Ltd., Shanghai, China), a customized Raman spectrograph with high-resolution volume phase holographic (P&P Optica Inc., Waterloo, ON, Canada) and a charge-coupled-device (CCD) camera (Andor-iDus-416, Oxford Instruments plc, Oxfordshire, UK). The software, HoloGRAMSTM (Ver. 3.2, Kaiser Optical Systems Inc., Ann Arbor, MI, USA) was used for calibration and spectral data acquisition.

**Figure 1:**
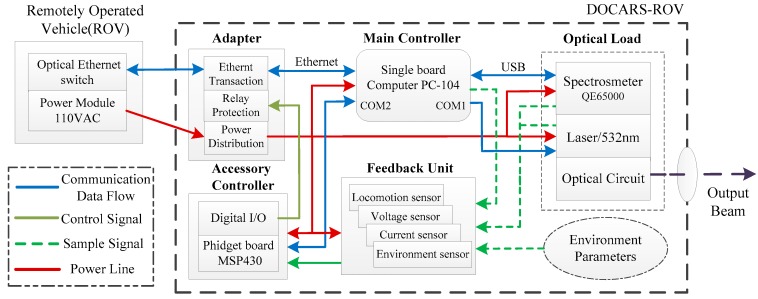
Schematic Diagram of DOCARS system.

In order to integrate all modules requiring diverse communication interfaces and power standards, DOCARS adopts a kind of dual-controller structure, as shown in Figure 1. In this structure, control functionality is shared by two diverse controllers that are mutually connected with a serial data bus. The main controller, PC-104 single board computer (PCM-3363), has an Ethernet interface and strong process capability. PCM-3363 is designed to communicate with the ship-based terminal and manipulate optical sensors. The auxiliary controller is designed based on microchip (MSP430). Compared with PCM-3363, MSP430 has higher reliability and strong resistance to Electromagnetic interference (EMI), which make it qualified for controlling underlying hardware.

In terms of system logic, the above configuration constitutes a layered structure whose communication channel has unidirectional order-flow and data-flow (spectral data and other sensor data). During the profile measurement process, Raman spectrogram and various sensor information (temperature, pressure, and humidity of the cabin) was collected, processed, and eventually uploaded to the ship-based terminal. In return, the ship-based terminal sends a variety of orders to the in situ measurement system, guaranteeing that the underwater system is operating on appropriate status. In this structure, the PCM-3363 and microchip board, together with the ship-based terminal, work as a distributed system entity. They operate different software respectively, to ensure operation of DOCARS system. The systematic control function is divided into two parts: optical sensors control and spectrum data process. 

**Figure 2:**
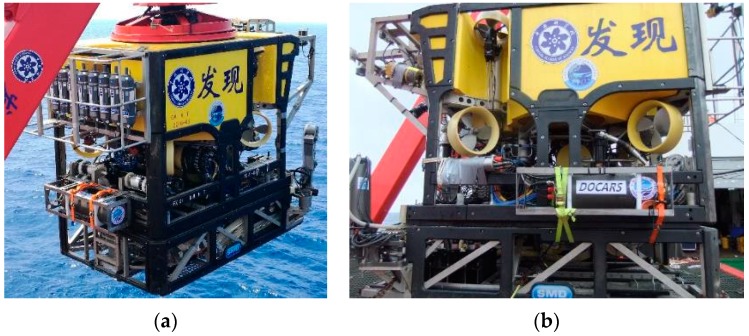
ROV installation solutions for DOCARS system. (**a**) Back mounting case. (**b**) Side mounting case.

Considering the application and maintenance cost of an ocean-oriented system, reliability is the first factor during design and installation. As in situ measurement system, DOCARS need to have enough resistance against the deep sea environment on its optical sensors. In practical sea-going research, DORCAS was powered and deployed in the field by the ROV Faxian. According to different measurement combination for ROV tasks, it can be installed on the different spots of ROV Faxian, as showed in Figure 2.

When DOCARS was mounted together with another laser induced breakdown measurement system (Libs-Sea) [7,10] that had a similar weight and dimensions as DOCARS, these two systems would be installed on two sides of the ROV to maintain structural balance. When DOCARS was mounted on ROV individually, it would be installed on the ROV back for the same reason. These arrangements have no difference during the profile measurement process, but will affect measurement results for targets on the seafloor (hydrothermal vent or other spots) because the back installation case laser spot of DOCARS is located out of the operation range of the ROV actuators and camera.

**Figure 3:**
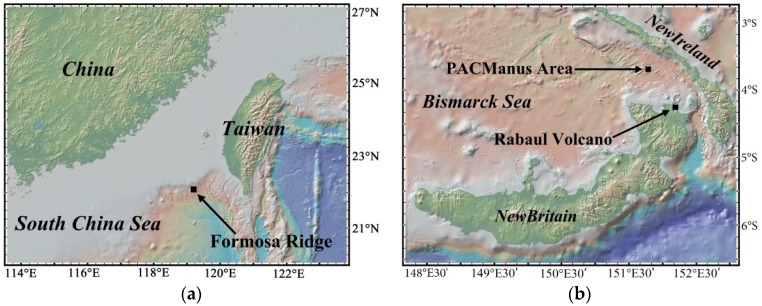
Map location of the sea-going area constructed with GeoMapApp (www.geomapapp.org). (**a**) Cold seep field at Formosa Ridge in the South China Sea [3]. (**b**) PACManus hydrothermal vent area at Paul Ridge in Bismarck Sea [1].

In order to analyze and model the profile measurement process, this article abstracts the sea-going research data collected in the PACManus hydrothermal area and the South China Sea; the mapping information is shown in Figure 3. The PACManus hydrothermal area is located at the crest of the Paul Ridge approximately 80 km off the Rabaul Volcano on the New Britain Arc [1]. The Paul Ridge is ~20 km long, 1–1.5 km wide and is characterized by an 800 m-long central neovolcanic zone. The Formosa Ridge is a 30-km-long, 5-km-wide submarine ridge with water depth around 1225 m located at the passive margin of the northeastern South China Sea [3].

In these spots ROV Faxian carrying the DOCARS system completed profile measurement three times (every time both including floating and diving parts), the detailed information of these processes is showed in Table 1. In each profile measurement we acquired seawater temperature recordings from the CTD of ROV and condition data (temperature, humidity, and pressure) inside DORCAS. Through appropriate modeling methodology we could predict the changing trend of cabin condition of DOCARS and then provide dependency for compensation and tuning algorithm for sophisticated optical sensors.

**Figure 4:**
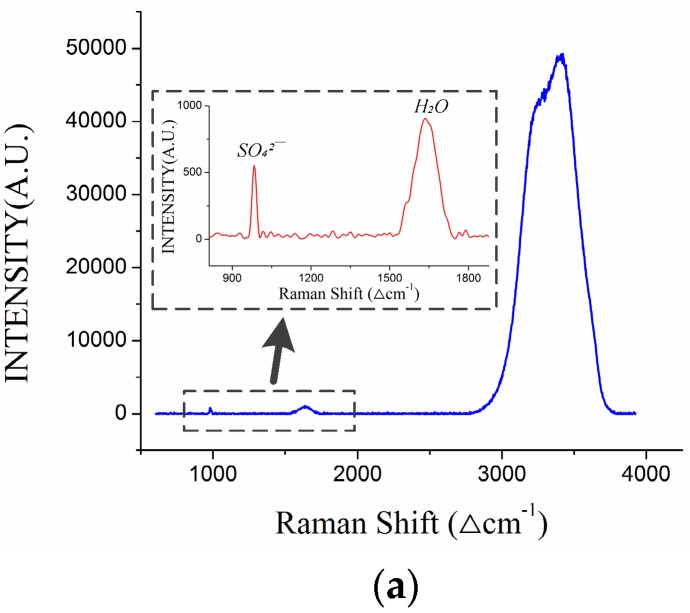

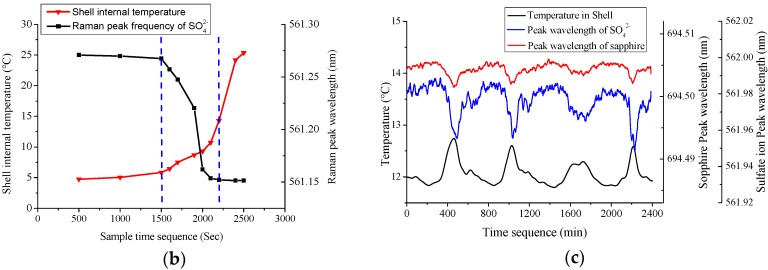
Effect of cabin temperature on Raman spectrum data. (**a**) Typical Raman spectrum and substance peaks. (**b**) Development trend of cabin temperature and SO_4_^2−^ Raman peak frequency (DOCARS system, #52 floating process). (**c**) Development trend among cabin temperature, Raman peak wavelength of sulfate ion and sapphire (OUC-Raman instrumental node).

As mentioned in the above section, the change in cabin condition may leave a negative effect on the optical devices in the DOCARS system, inducing a spectrum shift or laser power fluctuation [4]. The former is derived from the temperature drift of the camera and CCD, the latter corresponds to the laser output power [5]. In this paper, we mainly discuss the frequency shift of the Raman spectrum induced by temperature. Since different materials have different expansion characters in changeable conditions, rapid temperature decline during diving (or floating) could induce deformation of grating and then cause spectrum shift of optical sensors, such as the camera or CCD [18]. Typical Raman spectrum of seawater is shown in Figure 4a, the specific target substance corresponds to a unique peak in the Raman spectrum. Figure 4a is the typical Raman spectrum of the seawater sample, in this spectrum the H–O–H bending peak (~1640 ∆cm^−1^, corresponds ~583 nm in wavelength) of water and the S–O stretching peak of SO_4_^2-^ (~981 ∆cm^−1^, corresponds ~561 nm in wavelength) are evident. Figure 4b displays the development of SO_4_^2−^ and cabin temperature (#52, floating measurement process). It can be found that the Raman peak frequency of SO_4_^2−^ would decrease with increasing cabin temperature. It also can be found that the frequency shift range of the spectrum is not unbounded, the Raman peak of SO_4_^2−^ only decreased in a limited range (561.15–561.27 nm); out of this range it would tend to maintain regardless of the temperature change. In order to further demonstrate the effect of cabin temperature on the Raman frequency shift, data collected by the OUC-Raman instrumental node (applied in seafloor observatory network) [16,17] is employed in this section. Comparing to the ROV platform, the instrumental node of the seafloor network is located in a fixed seafloor position that has relatively stable water conditions. This enables us to exclude other factors’ effects on the substance Raman peak shift. Secondly, OUC-Raman node’s optical window is made up of sapphire material that also can be observed in the Raman spectrum. Since the sapphire window is hardly affected by other natural factors, its Raman peak can be used to evaluate the degree of frequency shift. One data sample (~40 h) of Raman spectrum sampled by the OUC-Raman system is shown in Figure 4c. It displays three parameters: temperature of cabin condition and the Raman peak frequency of the sapphire material and sulfate ion. In Figure 4c it can be found that Raman peak frequency of the sapphire and sulfate ion will decrease as cabin temperature increased. Since the temperature gradient inside the OUC-Raman system (~274 K) is far smaller than that inside DOCARS (~303 K), the Raman peak frequency kept pace with the temperature change in this small range.

The temperature-induced Raman frequency shift is one of obstacles for Raman quantitative analysis and automatic data process [19]. In order to solve this problem, there are two solutions; one is to build a relationship between the temperature factor and Raman frequency shift. The other solution is actively maintaining a steady state of cabin condition. The first solution is difficult to handle because this relationship is related to the multiple optical devices (laser, camera, CCD, etc.), it is hard to acquire a data compensation method with a general purpose [8]. Although the second solution is more feasible, it is still limited by power consumption (ROV is strict with its power management). It needs a measurement system that uses as little power for condition maintenance as possible. In order to realize the second solution, the controller of DOCARS should predict cabin condition change and make a moderate adjustment, which needs to have a full understanding towards the development model of cabin condition during the profile measurement process. 

## 3. Modeling for Profile Measurement Process

The entity of DOCARS is encapsulated with a customized 4000 m-class, 7075 aluminum alloy shell. Cabin temperature is one property of the cabin condition. In the profile measurement process, changeable seawater temperature (*T*_s_) could affect DOCARS’s cabin condition through shell conduction, which would induce its pressure (*P*) and temperature (*T*_c_) to change. Through comparing with tradition algorithms, it is found that the ARX (discrete autoregressive) model is an effective method to model the cabin condition and predict its development trend.

### 3.1. Temperature Prediction

Typically, the shell of the DORCAS system can be modeled as a cylinder of radius *R* and its thermal conductivity character stays constant during entire profile detection process, heat exchange between the cabin atmosphere and seawater will be abstracted as the radial heat conduction model [26]. The equation of this process is shown as Equation (1).

(1) $$\frac{\partial \theta }{\partial t}=\lambda \left(\frac{\partial^{2}\theta }{\partial t^{2}}+\frac{1}{r}\frac{\partial \theta }{\partial r}\right)\left(t>0,\;0<r<R\right)$$

In Equation (1), *θ* (*r*, *t*) is temperature of a specific point of the shell; it is a function of time t and distance from the shell center r. In order to solve Equation (1), traditional methods assume media (seawater) temperature is constant for the whole process [26]. Then, the above equation will be simplified and solved.

Although this simplification has been widely applied in multiple cases [25,27], it cannot be applied for the DOCARS system. The main obstacle is that the shell material of DORCAS is sandwiched between two media (cabin condition and outer seawater) and the temperature of these media both change during the profile measurement process. For this process it can hardly take numerical simulation using the above method [26]. A feasible solution is adopting a system identification method to fit the target process. Since the heat exchange efficiency between the cabin condition and seawater is relatively viscous, this article adopts a discrete autoregressive model (ARX) for system identification. In practical operation, sensors would sample *T*_s_ and *T*_c_ of the cabin condition synchronously, so the ARX model in this paper could ignore sampling delay time (*N*_k_) and be described as a set of differential expressions, as shown in Equations (2)–(4).

(2) A(z)y(t)=B(z)u(t)+v(t)

(3) $$A(z)=1+a_{1}z^{-1}+a_{2}z^{-2}+\ldots +a_{m}z^{-m}$$

(4) $$B(z)=1+b_{1}z^{-1}+b_{2}z^{-2}+\ldots +b_{n}z^{-n}$$

In Equations (2)–(4), *y*(*t*) is cabin temperature at time sequence *t*, and *u*(*t*) is temperature of seawater. Parameters m and n are orders of the numerator and denominator in ARX characteristic equations. *v*(*t*) stands for White-noise disturbance value.

An ARX-based predictive model is applicable to all finite-order systems with rational spectral density [29]. It also has low complexity and computation amount. These features make it suitable for embedding into the software of DOCARS. While DOCARS executes profile measurement, the microchip will monitor temperature, humidity, and pressure of the cabin condition and make gradient inversion with ARX model. If there is obvious deviation between the inversion result and expected value, the microchip will drive the relevant actuator to stabilize it.

When it comes to parameter estimation of the ARX model, the first step is to estimate the appropriate order of the model. This article uses Akaike’s information criterion (AIC) to calculate the system model order, and then estimates model parameters using the least square method (LSM) [30]. Since the sampling process of CTD (*T*_s_) and sensors inside the cabin (*T*_c_, *P*) are all stationary random processes, this guarantees that the estimation vector $\hat{\theta }(A,B)$ is an unbiased and consistent estimate of the real parameter vector θ(A,B).

**Figure 5:**
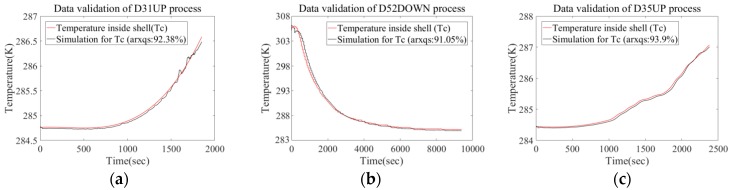
Comparison between real measurement and prediction data group (*T*_c_-*T*_s_). (**a**) Comparison for floating profile process of #31 ROV task. (**b**) Comparison for diving profile process of #52 ROV task. (**c**) Comparison for floating profile process of #35 ROV task.

**Figure 6:**
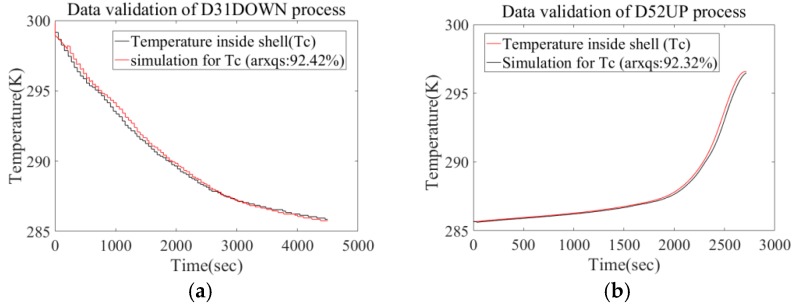
Comparison between real measurement and prediction data group (*T*_c_-*T*_s_). (**a**) Comparison for diving profile process of #31 ROV task. (**b**) Comparison for floating profile process of #52 ROV task.

In order to depress coupling of identification samples, we utilize the floating process of #31 task (D31UP), diving process of #52 task (D52DOWN), and diving process of #35 task (D35UP) for parameter estimation, then validate the result with the rest of the data sets. When generating a simulation output, the algorithm sets a 200 step (1 s per step) prediction horizon, because in application this time margin is enough for the ship-based terminal and actuator to respond. By comparing the prediction output with the measured data, we will validate the availability of the present ARX model. The comparison result is shown in Figure 5 and Figure 6. Based on data comparison in the above diagrams, it will be found there is no obvious difference in fitting degree between the validation set and identification set. Furthermore, data collected during #31 diving task did not start before ROV water entry, but at a depth of about 100 m. The fitting degree for this process (92.42%) indicates that the prediction calculation permits suspension or restart at a specific initial condition (*T*_c_, *T*_s_).

### 3.2. Pressure Prediction

Since cabin condition is sealed with shell material, the state of atmosphere of cabin condition primarily obeys Van der Waals equation, as shown in Equation (5).

(5) $$V_{m}^{3}-(b+RT/P)\cdot V_{m}^{2}+aV_{m}/P-ab/P=0$$

In Equation (5), parameters a, b are Van der Waals constants (pure nitrogen atmosphere, *a* = 1.36 L^2^·atm·mol^−2^, *b* = 0.04 L·mol^−1^), it could be further deducted into Equations (6) and (7).

(6) $$P/T=RT/\left(V_{m}-b\right)-\alpha$$

(7) $$\alpha =a/V_{m}^{2}\cdot T$$

**Figure 7:**
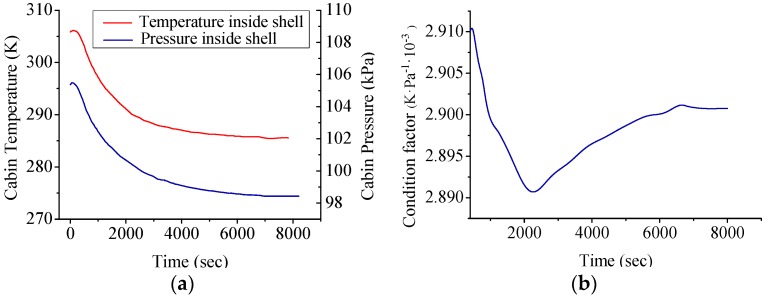
Development of cabin atmosphere during diving profile process of #52 ROV task. (**a**) Temperature (*T*_c_) and pressure (*P*) development trend inside cabin across time sequence *t*. (**b**) The ratio of *T*_c_ to *P*.

According to Van der Waals equation, we know the atmosphere of the cabin has a fairly constant ratio of temperature and pressure. However, by analyzing the measured development trend of cabin temperature (*T*_c_) and pressure (*P*) in a time series, especially combining with CTD data of ROV, we find that the ratio of *T*_c_ and *P* of the cabin’s atmosphere was fluctuating throughout the whole profile measure process and tended to converge after ROV reached the seabed, shown in Figure 7.

**Figure 8:**
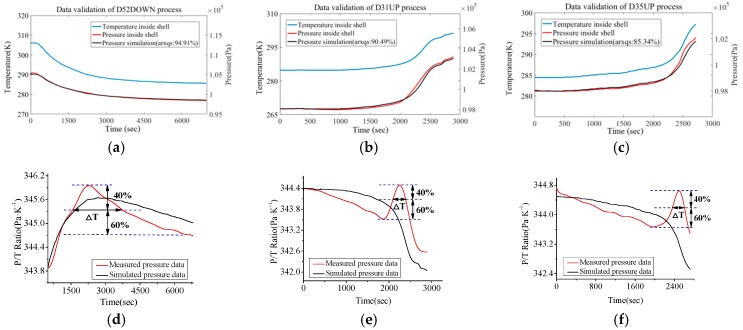
Data validation between real measurement group and predicted simulation group (*T*_c_-*P*). (**a**) Comparison for diving profile measurement process of #52 ROV task. (**b**) Comparison for floating profile measurement process of #31 ROV task. (**c**) Comparison for floating profile measurement process of #35 ROV task. (**d**) Trend of *P*/*T*_c_ during diving profile process of #52 ROV task. (**e**) Trend of *P*/*T*_c_ during floating profile process of #31 ROV task. (**f**) Trend of *P*/*T*_c_ during floating profile process of #35 ROV task.

**Figure 9:**
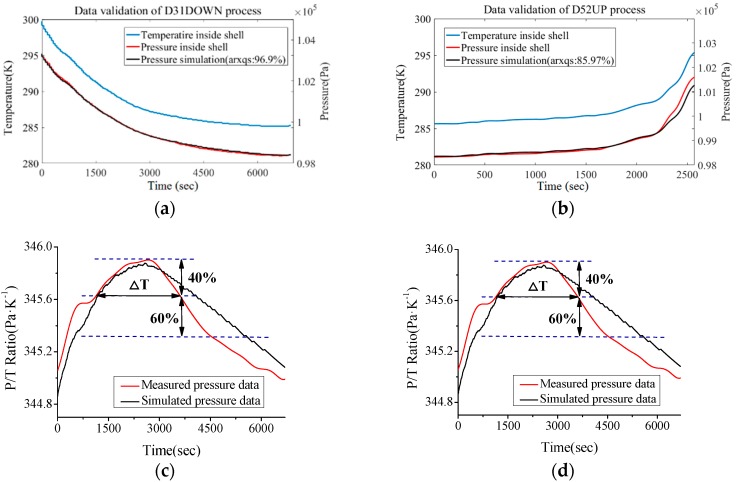
Data validation between real measurement group and predicted simulation group (*T*_c_-*P*). (**a**) Comparison for diving profile measurement process of #31 ROV task. (**b**) Comparison for floating profile measurement process of #52 ROV task. (**c**) Trend of *P*/*T*_c_ during diving profile process of #31 ROV task. (**d**) Trend of *P*/*T*_c_ during floating profile process of #52 ROV task.

In Figure 7 it was found that cabin temperature continuously decreased as the ROV dived down (cooling effect of seawater), which indirectly affected the state of the cabin atmosphere. Moreover, the follow-up mode of *T*_c_ and *P* is not strictly synchronous (ratio of these two parameters show a damped oscillation trend). Similarly, as discussed in the above content, it is intended to describe this trend with a discrete dynamic model (ARX model) instead of the Van der Waals equation. In these recording processes the condition maintenance function of DOCARS was turned off, so we could investigate the natural transition of the atmosphere state inside DOCARS. For the sake of building the atmosphere condition model of DOCARS, it takes a similar estimation and validating mechanism as in the last chapter, utilizing the floating process of #31 task (D31UP), diving process of #52 task (D52DOWN), and floating process of #35 task (D35UP) for order estimation (based on AIC) and parameter estimation (based on LSM), then to validate the result with the rest of data sets (D52UP and D31DOWN). Comparison between simulation output and measured data is showed in Figure 8 and Figure 9.

The validation result indicates that the ARX model has a higher fitting level on the diving process (average fitting is more than 90%) than that on the floating process (has an average fitting degree more than 85%). This trend also could be found by validating the ratio of cabin temperature (*T*_c_) and pressure (*P*). Through the *P*/*T* curve it is found that in the floating process the trend of the ratio of *T*_c_ and *P* is relatively steady and well-traced by the predictive curve most of the time, but in the end of the floating process there is a peak that the predictive curve does not trace the real process well. The ROV log indicates this peak took place when the DORCAS was floating across the thermocline layer in which the seawater temperature changes the most drastically in the entire profile range [22]. It is observed that the peak of the ratio is closely related to floating and diving speed of ROV (actually it is the temperature changing speed ∆*T*_s_/∆*t*), as shown in Table 2.

From what is shown in Table 2 the relationship between *T*_c_/*P* peak and ∆*T*_s_/∆*t* can be built. When ROV is diving or floating across the thermocline layer, the *T*_c_/*P* curve of DORCAS will produce obvious peak distortion, the degree of such distortion is directly proportional to temperature gradient. On the other hand, the present fitting percentage is enough for engineering applications, if the temperature gradient is kept under 5.0 × 10^−3^ K/s (like that of D52DOWN process), the simulated *T*_c_/*P* ratio will match the actual well. 

Through this ARX model, the existing controller inside DOCARS will make use of condition sensors (temperature, humidity, and pressure) to inverse and predict the gradient of the cabin condition during diving or floating processes. The error between prediction and expectation will be regarded as dependency on the temperature control or data compensation algorithm. 

## 4. Conclusions

This paper describes the design of the DOCARS measurement system and strategy for its cabin condition maintenance. The DORCAS system is specially designed to mount on a ROV platform for deep sea in situ measurement. It is developed to collect and analyze chemical distribution of seawater. This paper discusses that during profile measurement processes the cabin temperature of the system will cause a frequency shift of the Raman spectrum. According to measurement data acquired from multiple measurement systems (DOCARS of ROV and OUC-Raman of seafloor observatory network), it is found that the Raman peak frequency of the target substance will experience a shift as the cabin temperature changes. That will affect the quantitative analysis and automatic process for the Raman spectrum data. In order to solve this problem, it needs to maintain a stable cabin condition of DOCARS. In this paper, we propose a modeling methodology to predict the change trend of cabin temperature and pressure during the profile measurement process. A set of data abstracted from sea-going researches is utilized to validate this methodology. Through analysis on the system model and profile measurement process, a predictive ARX model (200 steps ahead) is adopted and shows a good fitting degree for real development trends (*T*_c_-*P*, *T*_c_-*T*_s_) of DOCARS. A criterion for this predictive model is also derived in terms of the temperature gradient across a thermocline layer.

The ARX model was trained by limited sea-going samples to build the heat-transfer character of the deep-sea measurement system, but the trained ARX model also had a good predictive result for other sea-going samples (as shown in Figure 6 and Figure 9). In the next stage of this research, efforts will be taken to design a temperature controlling module for DOCARS system. Since the trained ARX has good applicability, its simulation data generated by the ARX model can help to validate the effectiveness of the temperature controlling module. This will increase the designing efficiency and decrease the experimental cost. On the other hand, although the ARX-based model provides practical guidance to maintain stable cabin condition of the DOCARS system, there exist some issues that have to be improved to make it more useful.

Firstly, the ARX-based predictive model needs further validation and optimization, especially its validity in more capricious and extreme underwater conditions. In terms of the model, ∆*T*_s_/∆*t* is both related to actual thermal distribution across the profile, seawater, and moving speed of ROV. Some subsection optimization could be considered to suppress the deviation between the real and predicted trend. 

Secondly, considering that the temperature and pressure of the cabin (*T*_c_, *P*) are parallel collected, this paper ignores the sample delay between seawater temperature and cabin temperature. Seawater temperature data comes from the CTD sensor of ROV; there exists sample delay (*N*_k_) between the *u*(t) and *y*(t) in the ARX model (*T*_s_-*T*_c_). Although the sampling rate greatly outweighs the temperature changing trend, it also could try to add the *N*_k_ into the ARX estimation to acquire optimized prediction performance.

## Figures and Tables

**Table 1:** Information of profile measurement process.

Index	Investigation Area	Profile Depth (m)
#31	PACManus hydrothermal field, Paul Ridge	1731
#35	PACManus hydrothermal field, Paul Ridge	2180
#52	South China Sea, Formosa Ridge, cold seep site	1170

**Table 2:** Information of profile measurement process.

Diving ID	Time to across Thermocline ∆t (s)	Temperature Shift ∆*T*_s_ (K)	∆*T*_s_/∆*t* (10^−3^ K/s)
D31DOWN	2358	11.32	4.80
D31UP	326	17.03	52.23
D35UP	361	14.96	41.44
D52DOWN	2164	15.86	7.32
D52UP	317	20.183	63.67
